# Glutamine and Alanyl-Glutamine Increase RhoA Expression and Reduce *Clostridium difficile* Toxin-A-Induced Intestinal Epithelial Cell Damage

**DOI:** 10.1155/2013/152052

**Published:** 2012-12-27

**Authors:** Ana A. Q. A. Santos, Manuel B. Braga-Neto, Marcelo R. Oliveira, Rosemeire S. Freire, Eduardo B. Barros, Thiago M. Santiago, Luciana M. Rebelo, Claudia Mermelstein, Cirle A. Warren, Richard L. Guerrant, Gerly A. C. Brito

**Affiliations:** ^1^Department of Morphology, Faculty of Medicine, Federal University of Ceará, Delmiro de Farias, 60416-030 Fortaleza, CE, Brazil; ^2^Department of Physiology and Pharmacology, Faculty of Medicine, Federal University of Ceará, 1127 Coronel Nunes de Melo, 60430-270 Fortaleza, CE, Brazil; ^3^Department of Physics, Faculty of Physics, Federal University of Ceará, 922 Campus do Pici, 60455-760 Fortaleza, CE, Brazil; ^4^Biomedical Sciences Institute, Federal University of Rio de Janeiro, 373 Avenue Carlos Chagas, 21941-902 Rio de Janeiro, RJ, Brazil; ^5^Division of Infectious Diseases and International Health, Center for Global Health, University of Virginia, 345 Crispell Drive, Room 2709, Charlottesville, VA 22903, USA

## Abstract

*Clostridium difficile* is a major cause of antibiotic-associated colitis and is associated with significant morbidity and mortality. Glutamine (Gln) is a major fuel for the intestinal cell population. Alanyl-glutamine (Ala-Gln) is a dipeptide that is highly soluble and well tolerated. IEC-6 cells were used in the *in vitro* experiments. Cell morphology was evaluated by atomic force microscopy (AFM) and scanning electron microscopy (SEM). Cell proliferation was assessed by WST-1 and Ki-67 and apoptosis was assessed by TUNEL. Cytoskeleton was evaluated by immunofluorescence for RhoA and F-actin. RhoA was quantified by immunoblotting. TcdA induced cell shrinkage as observed by AFM, SEM, and fluorescent microscopy. Additionally, collapse of the F-actin cytoskeleton was demonstrated by immunofluorescence. TcdA decreased cell volume and area and increased cell height by 79%, 66.2%, and 58.9%, respectively. Following TcdA treatment, Ala-Gln and Gln supplementation, significantly increased RhoA by 65.5% and 89.7%, respectively at 24 h. Ala-Gln supplementation increased cell proliferation by 137.5% at 24 h and decreased cell apoptosis by 61.4% at 24 h following TcdA treatment. In conclusion, TcdA altered intestinal cell morphology and cytoskeleton organization, decreased cell proliferation, and increased cell apoptosis. Ala-Gln and Gln supplementation reduced intestinal epithelial cell damage and increased RhoA expression.

## 1. Introduction

*Clostridium difficile *(*C. difficile*), a gram-positive bacillus, is considered the most frequent cause of diarrhea associated with the use of antibiotics in industrialized countries and is considered a major challenge among hospitalized patients exposed to long-term antibiotic treatment resulting in increased morbidity, mortality, and length of hospitalization [1–4]. Studies from the US, Canada, and the European Union have reported increased numbers of cases of *Clostridium difficile* infection (CDI) [5, 6]. Furthermore, recent studies have demonstrated an increase in disease severity and case-fatality rates [6–10], associated with the emergence of a more virulent strain-NAP1/B1/027, that carries a binary toxin (CDT) and produces elevated quantities of A toxin (TcdA) and B toxin (TcdB) and increased numbers of spores [9, 11].

TcdA and TcdB have glucosyltransferase activity and lead to disaggregation of actin by inactivation of Rho [12, 13]. Recently, using a hamster model of infection, it has been demonstrated that either TcdA or TcdB alone, produced by isogenic mutants of *C. difficile, *may cause severe disease [14]. Additionally, similar results were found when either gene was permanently inactivated using a gene knockout system. Finally, virulence was completely attenuated when both genes were inactivated, highlighting the importance of both TcdA and TcdB [14].

TcdA induces monoglycosylation of Rho, Cdc42, and Rac,which inhibits the Rho family proteins role in the formation of actin filaments, leading to cellular restrain, loss of adhesion, and cell rounding [15–18]. Nam et al. [19] demonstrated that TcdA causes microtubule depolymerization by tubulin deacetylation through activation of HDA6, which is involved in cytokine production, alpha-tubulin deacetylation, and mucosal damage. TcdA also causes intestinal secretion, intense destruction of the mucosa, hemorrhage, and accentuated inflammation with neutrophil infiltration and production of inflammatory cytokines such as TNF-*α* and IL1-*β* [20, 21]. Additionally, TcdA induces cellular rearrangement of actin cytoskeleton into aggregates and increases secondary adhesion, by inducing Mac-1 expression in human neutrophils. Such events could be associated with the formation of pseudomembranes [22, 23]. 

Glutamine (Gln) is the major respiratory fuel for the intestinal epithelium, since it is a precursor for nucleotide biosynthesis and, therefore, a critical requirement for the dynamic proliferating intestinal cell population. However, glutamine has limited solubility and a tendency to hydrolyze to potentially toxic glutamate. It has been demonstrated that alanyl-glutamine (Ala-Gln) is stable, highly soluble, well tolerated, and at least as effective in driving sodium cotransport and intestinal injury repair *in vitro* [23–26] in animals [27] and in patients [28]. Glutamine supplementation influences inflammatory response, oxidative stress, apoptosis modulation, and the integrity of gut barrier [28]. Carneiro et al., 2006 [26] demonstrated that Gln and Ala-Gln significantly reduced the intestinal damage caused by TcdA in rabbit ileal loops and the amount of intestinal epithelial cell apoptosis.

In this study, we evaluated the effects of Gln or Ala-Gln supplementation on intestinal epithelial cell injury induced by TcdA.

## 2. Materials and Methods

### 2.1. Reagents, Drugs, and Toxin

Trypsin, Dulbecco's modified Eagle media (DMEM), fetal bovine serum (FBS), RPMI media, penicillin-streptomycin, sodium pyruvate, and antibiotic antimycotic solution were obtained from either Gibco BRL (Grand Island, NY, USA) or Invitrogen (Carlsbad, CA). Gln, Ala-Gln, and TcdA of *C. difficile* (c3977), tetrazolium salt WST-1 (4-[3-(4-iodophenyl)-2H-5-tetrazolio]-1-3-benzene disulfonate), bovine insulin, DAPI- and FITC-conjugated anti-mouse secondary antibodies were obtained from Sigma (St. Louis, MO, USA). Anti-RhoA monoclonal mouse primary antibody (Santa Cruz Biotechnology, CA, USA). 

### 2.2. Cell Culture

Rat intestinal jejunal crypt cells (IEC-6, passages 7–24) were purchased from American Type Culture Collection (Rockville, MD, USA) and cultured at 37°C in a 5% CO_2_ incubator. When 90–95% confluency, cells were trypsinized with 0.25% EDTA trypsin. Cells were cultivated in 75 cm^2^ flasks, and media were changed twice a week. For IEC-6 cells, the maintenance cell medium was DMEM (Gibco BRL, Grand Island, NY, USA) supplemented with 5% FBS, 5 mg bovine insulin, 50 *μ*g/mL of penicillin/streptomycin (Gibco BRL, Grand Island, NY, USA), and a final concentration of 1 mM of sodium pyruvate. The medium was changed thrice a week, according to standard culture protocols [24, 26].

### 2.3. Atomic Force Microscopy

In order to evaluate the effect of TcdA in IEC-6 cell morphology by atomic force microscopy (AFM), 12-well cell culture plates, with 13 mm diameter glass coverslips, were seeded with 6.25 × 10^4^ IEC-6 cells and grown for 24 h in standard DMEM media. Then, the wells were washed and incubated for 1 h with TcdA (100 ng/mL) in standard DMEM. Since TcdA at 100 ng/mL caused severe damage on IEC-6 cell morphology, we used 10 ng/mL to evaluate the protective effect of Gln and Ala-Gln. For this, 12-well cell culture plates, with 13 mm diameter glass coverslips, were seeded with 6.25 × 10^4^ IEC-6 cells and grown for 24 h in standard DMEM media. Then, the wells were washed and incubated for 24 h with TcdA without Gln or supplemented with 10 mM of Ala-Gln or 10 mM of Gln. Afterwards, cells were fixed to glass coverslips in 4% formaldehyde solution for 14 h. For the imaging process, the samples were air-dried for 5 min, placed on steel sample disks covered with doublesided adhesive tape and carried off to Multimode Atomic Force Microscope (Digital Instruments, Santa Barbara, CA, USA) equipped with a NanoScope IIIa controller. Scans were performed in air, and all topography images were acquired by contact mode using silicon crystal cantilevers (Veeco-probes) with a spring constant of approximately 40 N/m and tip radius of 15 nm. The AFM height data was represented as a distinct height value of the sample in a finite number of pixels (512 × 512 point scan) [29]. The clearest regions indicate the highest area, which in the control cell indicates the localization of the nucleus. All topography images were performed with a Nanoscope IIIa controller and NanoScope software (Digital Instruments, CA, USA) at room temperature. The area, height, and volume of the cells were calculated using NanoScope 5.30 R3.SR3. The volume was calculated using bearing tool, in which the calculation is performed through the volume of a set of pixels bounded by second planes [29, 30]. 

### 2.4. Scanning Electron Microscopy

Twelve-well cell culture plates, with 13 mm diameter glass coverslips, were seeded with 6.25 × 10^4^ IEC-6 cells and grown for 24 h in standard DMEM media. Afterwards, the wells were washed and incubated for 24 h with TcdA (10 ng/mL) in DMEM without Gln or supplemented with 10 mM of Ala-Gln or 10 mM of Gln. Cells were than fixed in 4% formaldehyde for 14 h. For imaging, the samples were fixed to samples holders with carbon adhesive tape and sputtered with a 15 nm gold layer (BALTEC MED 020 coating system) and transferred into the scanning electron microscope (TESCAN VEGA-XMU) [31, 32]. 

### 2.5. Immunofluorescence Microscopy and Digital Image Acquisition

Six-well cell culture plates were seeded with 6 × 10^5^ IEC-6 cells and grown for 48 h in standard DMEM media (which contains Gln). Wells were washed and incubated for 24 h with TcdA (10 ng/mL) in DMEM without Gln or supplemented with 10 mM of Ala-Gln or 10 mM of Gln. IEC-6 were rinsed with PBS and fixed with 4% paraformaldehyde in PBS for 10 min at room temperature. They were then permeabilized with 0.5% Triton-X 100 in PBS for 30 min. The same solution was used for all subsequent washing steps. Cells were incubated with anti-RhoA monoclonal mouse primary antibody for 1 h at 37°C. After incubation, cells were washed for 30 min and incubated with FITC-conjugated mouse secondary antibody for 1 h at 37°C. After incubation, cells were washed for 30 min and incubated with Rhodamine-phalloidin for 30 min at 37°C. Afterwards, cells were incubated with DAPI (0.5 ug/mL) diluted in 0.9% NaCl for 5 min at 37°C and washed once with 0.9% NaCl. Cells were mounted in ProLong Gold antifade reagent (Molecular Probes) and examined with an Axiovert 100 microscope (Carl Zeiss, Germany) by using filter sets that were selective for each fluorochrome wavelength channel. Images were acquired with a C2400i integrated charge-coupled device camera (Hamamatsu Photonics, Shizuoka, Japan) and an Argus 20 image processor (Hamamatsu). Control experiments with no primary antibodies showed only faint background staining (supplementary material) [33].

### 2.6. Polyacrylamide Gel Electrophoresis and Immunoblotting

Six-well cell culture plates were seeded with 6 × 10^5^ IEC-6 cells and grown for 48 h in standard DMEM media (which contains Gln). Wells were washed and incubated for 24 h with TcdA (10 ng/mL) in DMEM without Gln or supplemented with 10 mM of Ala-Gln or 10 mM of Gln. Cells were quickly washed in ice-cold PBS and 50 mL of sample buffer (4% sodium dodecyl sulphate—SDS, 20% glycerol, 0.2 M dithioethreitol, 125 mM Tris-HCl, pH 6.8) were added to the cells and boiled for 5 min. Samples were loaded in 12% SDS-polyacrylamide gels (SDS-PAGE) and transferred to PVDF membranes. Then, the PVDF membranes were incubated overnight with anti-RhoA monoclonal mouse antibody. Membranes were washed thrice with TBS-T and incubated for 1 h with peroxisome-conjugated goat anti-mouse secondary antibody. Finally, membranes washed again as described above, and the bands were visualized using the ECL plus Western Blotting Detection System (Amersham). To check sample loading, another PVDF membrane (containing the same samples in the same volume used for the other blots) was incubated with a mouse monoclonal anti-alfa-tubulin antibody (dilution 1 : 3000 in TBS-T). After three washes in TBS-T (3 min each), the membrane was incubated with a peroxidase-conjugated goat anti-mouse antibody (dilution 1 : 7000 in TBS-T) and developed as described above. Quantification of protein bands was performed using the public domain software ImageJ (http://rsb.info.nih.gov/ij/) with data obtained from two independent experiments [33].

### 2.7. Proliferation Assay

IEC-6 cells were seeded in 96-well plates at the concentration of 10^5^ cells per well and allowed to grow O/N until 80% of full confluence. Next day cells were washed with PBS and challenged for 24 and 48 h with TcdA at 100 ng/mL, 10 ng/mL, 1 ng/mL, and 0.1 ng/mL with or without Ala-Gln (Sigma, St. Louis, MO, USA), at 10 mM, diluted in Gln-free medium. Ala-Gln was used to evaluate cell apoptosis and proliferation since it is more stable than the Gln and similar results when compared to Gln in the morphological analysis. Cultured cells, not challenged with TcdA, served as controls. Cell proliferation reagent WST-1 (10 *μ*L; Roche, Indianapolis, IN, USA) was added 24 and 48 hours after the treatment into 96-well plates to measure metabolic activity of viable cells. We incubated cells with WST-1 for 2 hours in controlled humidified chamber. During that time, viable cells convert WST-1 to a water-soluble formazan dye. Absorbances were measured at 450 nm using ELISA plate reader (BioTek Instruments Inc.). The absorbance directly correlates with cell number [23, 24].

### 2.8. TUNEL Assay

IEC-6 cells were plated in 2-well tissue culture chamber slides at a concentration of 10^5^ cells per well. Cells were allowed to attach the chamber slide for 24 hours. Cells were then exposed for 24 h with TcdA 10 ng/mL and Ala-Gln 10 m M. After the treatment, cells were washed trice with 1xPBS and fixed in 4% paraformaldehyde (methanol-free) for 15 minutes, then washed trice with 1xPBS. Cells were stained with using DeadEnd Fluorometric TUNEL kit from (Promega, Madison, WI, USA). TUNEL System measures the fragmented DNA of apoptotic cells by catalytically incorporating fluorescein-12-dUTP at 3′-OH DNA ends using the enzyme TdT (terminal deoxynucleotidyl transferase). Apoptotic cells were visualized using DAPI (SouthernBiotech, Birmingham, AL, USA). Images were taken under 20X magnification using Fluorescent Olympus 1 × 71 Inverted microscope with QImaging camera, with QCapture Pro.5.1 software. We evaluated 5–8 randomly selected fields. Cells were manually counted per selected area and expressed in percentage of apoptotic cells per mm^2^.

### 2.9. Ki67 Immunohistochemistry

Ki67 is a nuclear protein that is tightly linked to the cell cycle. It is a marker of cell proliferation. Ki67 is expressed in proliferating cells during mid-G_1_ phase, increasing in level through S and G_2_, and peaking in the M phase of the cell cycle [34]. IEC-6 cells were plated in 2-well tissue culture chamberslides at a concentration of 10^5^ cells per well. Cells were allowed to attach to the chamberslide for 24 hours. After 24 hours, cells were washed with PBS and treated with different concentrations of TcdA (100 ng/mL, 10 ng/mL, 1 ng/mL, and 0.1 ng/mL) and 10 mM Gln/Ala-Gln After the 24 hours of treatment, cells were washed trice with 1xPBS and fixed in 4% paraformaldehyde (methanol free) for 15 minutes, then washed trice with 1xPBS. IEC-6 cells were stained by using Ki67 antibody (MKI67rabbit monoclonal primary antibody) from Epitomics Inc., (Burlingame, CA, USA). Working dilution of primary antibody was 1 : 400. Secondary antibody used in procedure was anti-rabbit from DAKO, (Carpinteria, CA, USA). Staining was done in Tissue Research Core Facility at University of Virginia Medical School. Images were taken in the Core Facility using Olympus DP71 microscope and Microsuite Pathology Edition software using 20X magnifications with a 100 *μ*m scale. We evaluated 6 to 8 randomly selected areas. Ki67-positive cells were counted manually and calculated per total number of cells, expressed as percentage of Ki67-positive cells [34].

### 2.10. Statistical Analyses

 Results are expressed as mean ± standard error (SEM) using GraphPad Prism version 5.0 (GraphPad software, San Diego, CA, USA). Either one-way ANOVA, with Bonferroni's posttest, or unpaired Student's *t*-test were used to compare the differences between the experimental groups. Statistical significance was accepted at the level of *P* < 0.05.

## 3. Results

### 3.1. Effect of TcdA on IEC-6 Morphology and the Effect of Gln and Ala-Gln Treatment on Cellular Morphology and Dimensions as Evaluated through AFM

**Figure 1:**
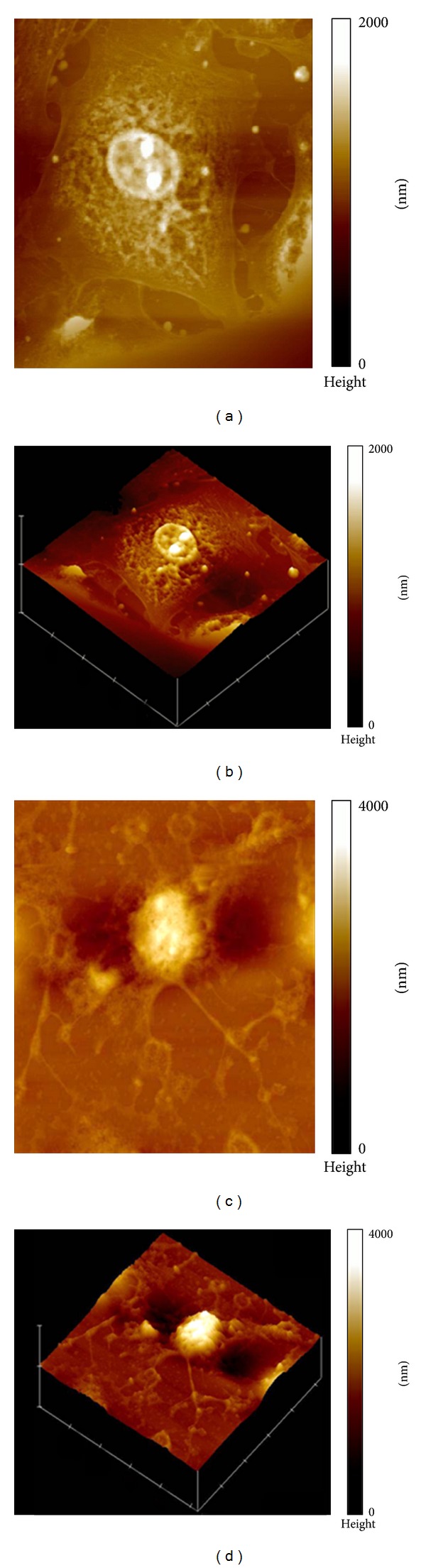
Atomic force microscopy (AFM) analysis of IEC-6 normal cell morphology with height scale (color bar): 2000 nm (a) and image of normal IEC-6 cell in 3D view (b). Effect of 1 h exposure with TcdA (100 ng/mL) on IEC-6 cell morphology with height scale (color bar): 4000 nm (c) and image of IEC-6 cell treated with TcdA (100 ng/mL) in 3D view (d). The images were obtained using AFM in contact mode (scan size 50 × 50 *μ*m^2^).

**Figure 2:**
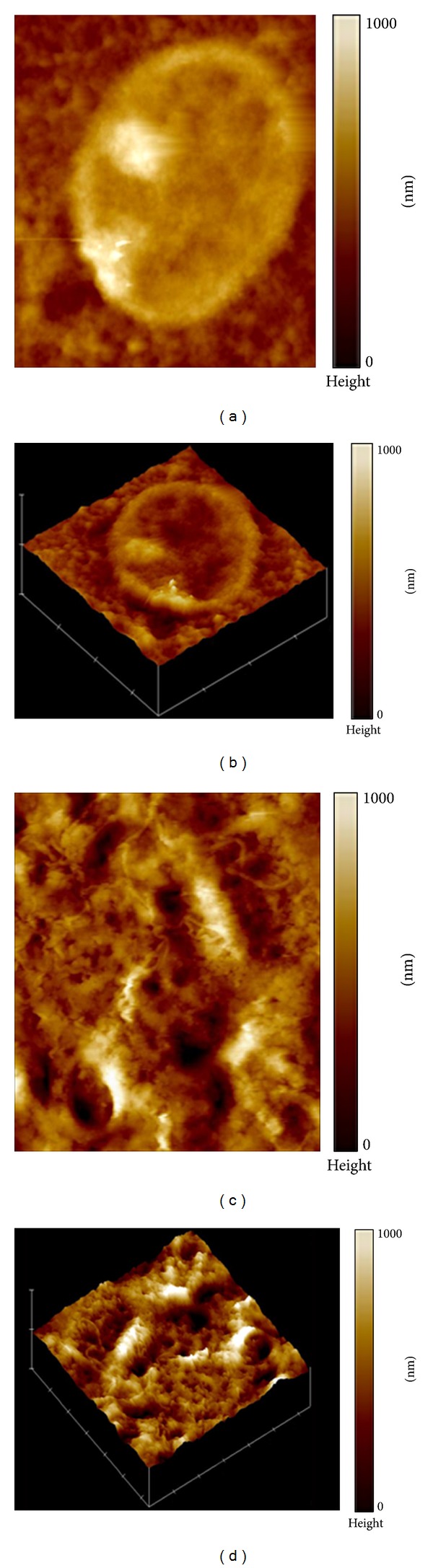
Atomic force microscopy (AFM) analysis of IEC-6 cell nucleus shape with height scale (color bar): 1000 nm (a) and image of normal IEC-6 cell nucleus in 3D view (b). Effect of 1 h exposure with TcdA (100 ng/mL) on IEC-6 cells nucleus morphology with height scale (color bar): 1000 nm (c) and image of IEC-6 cell treated with TcdA (100 ng/mL) in 3D view (d). The images were confectioned with AFM in the contact mode (15 × 15 *μ*m^2^).

**Figure 3:**
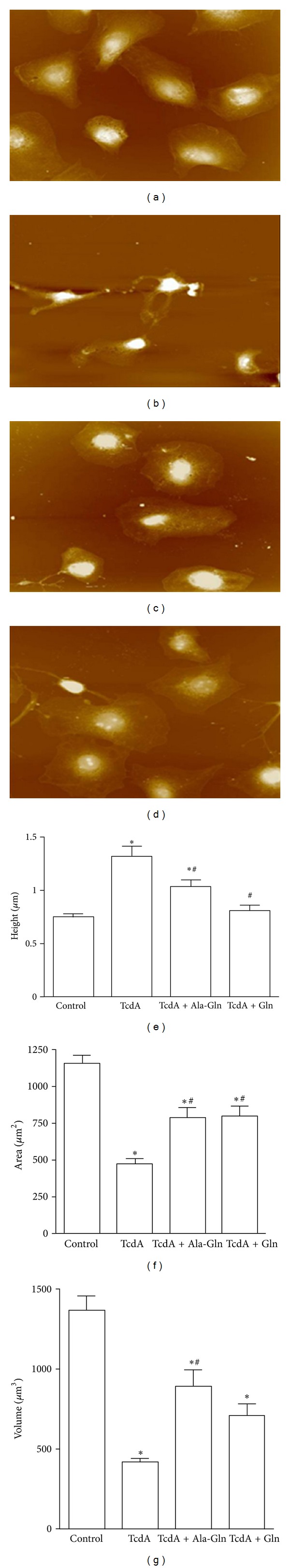
Atomic force microscopy analysis of the effects of glutamine and alanyl-glutamine supplementation on IEC-6 cell morphology following damage induced by TcdA. Effect of alanyl-glutamine (TcdA + Ala-Gln) or glutamine supplementation (TcdA + Gln), both at 10 mM, simultaneously with 24-hour exposure to TcdA at 10 ng/mL on IEC-6 cell. The images were confectioned with AFM in the contact mode with scanning of 150 × 150 *μ*m^2^. Control (a), TcdA (b), TcdA + Ala-Gln (c), and TcdA + Gln (d). The height (e), the area (f), and volume (g) were calculated with the nanoscope 5.30R3.SR3 software. *P < 0.05 compared to control. ^#^ *P* < 0.05 compared to TcdA.

IEC-6 cells grown in normal media displayed well-preserved cytoplasm, nucleus, and nucleoli (Figures 1(a) and 1(b)). Treatment with TcdA caused shrinking and compression of cytoplasmic material around the nucleus, blurring of the nuclear membrane, and condensation of nuclear elements (Figures 1(c) and 1(d)). Multiple vestigial filamentous extensions around the pyknotic cell were observed. In the presence of TcdA, the nucleus height of a representative IEC-6 cell was increased to 4000 nm (Figures 1(c) and 1(d)), compared to 2000 nm in the control group (Figures 1(a) and 1(b)). Visualization of the nucleus by AFM at higher magnification showed unchallenged IEC-6 cell nucleus to have well-defined nuclear envelope and prominent nucleoli (Figures 2(a) and 2(b)). The TcdA challenged cell had complete disruption of the nuclear envelope, condensation of chromatin, and loss of the nucleolar apparatus (Figures 2(c) and 2(d)). Measurement of cellular dimensions revealed TcdA-challenged IEC6 cells to have a 58.9% increase in cell height (Figure 3(e)), which may indicate deposition of cytoplasmic material in the nuclear region. However, cell area and cell volume were noted to be decreased by 66.2% and 79%, respectively, compared to control (Figures 3(f) and 3(g)) (*P* < 0.05). Supplementation with 10 mM of Gln caused an increase of 46.3% and 67.6% in cell volume and area, respectively, and a reduction of 46.3% in the cell height in relation to the group treated with TcdA (*P* < 0.05, Figure 3). Supplementation with 10 mM of Ala-Gln significantly increased cell volume and area by 92.9% and 65.4%, respectively, and decreased cell height by 16.9% (*P* < 0.05 compared to TcdA-treated group). 

### 3.2. Effect of TcdA on Rat Intestinal Epithelial Cell (IEC-6) Morphology and the Prevention of Injury by Gln and Ala-Gln through SEM

**Figure 4:**
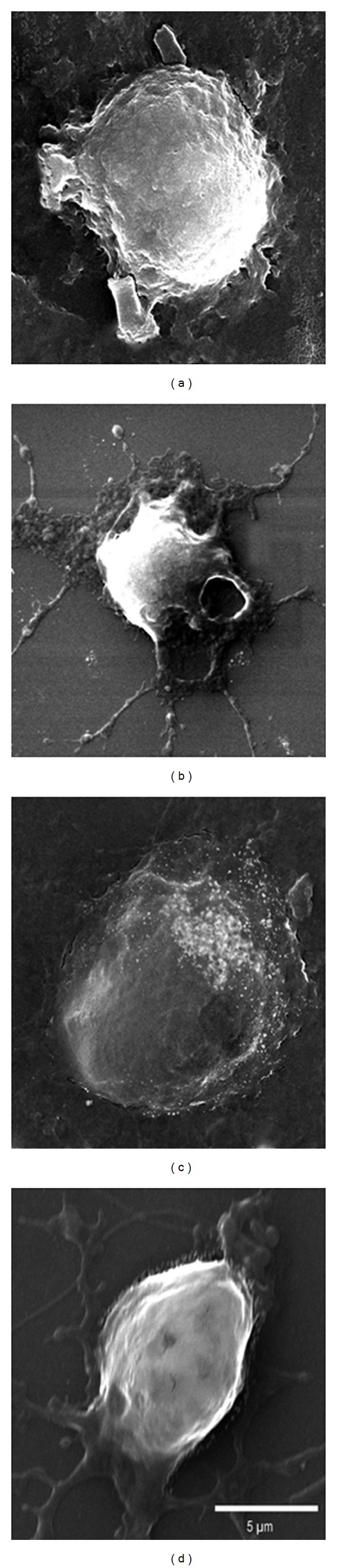
Electromicroscopy analysis of the effects of glutamine or alanyl-glutamine supplementation on IEC-6 cells following damage induced by *Clostridium difficile's* TcdA. The IEC-6 cells were divided into 4 groups: control (a), treated with TcdA at 10 ng/mL for 24 h (b), treated with TcdA at 10 ng/mL for 24 h and supplemented with 10 mM Ala-Gln (c), treated with TcdA at 10 ng/mL for 24 h and 10 mM of Gln (d). The images were confectioned with SEM, magnification 9.45X and scale bar 5 *μ*m.

Consistent with AFM findings, SEM also demonstrated shrinkage of IEC-6 cells in the presence of TcdA. Similarly, just a few thin cytoplasmic extensions or projections around the pyknotic body are left compared to the plump appearance of healthy cells in the absence of TcdA (Figure 4(b)). The cells treated with TcdA in medium contain 10 mM of Ala-Gln (Figure 4(c)) or Gln (Figure 4(d)) showed partial preservation of cell morphology displaying a “pancake-like” shape similar to control cell incubated in medium without TcdA (Figure 4(a)). 

### 3.3. Effect of TcdA and Ala-Gln on Cell Apoptosis

**Figure 5:**
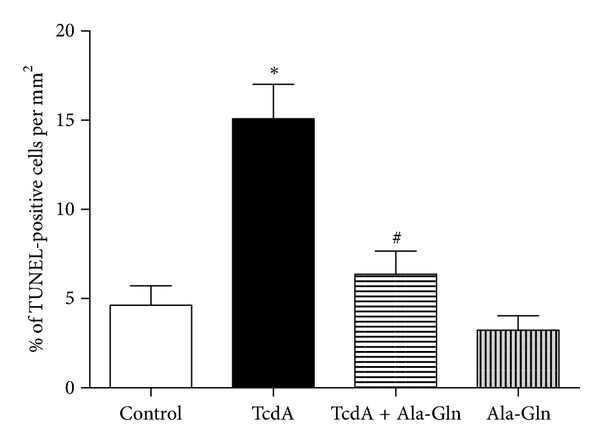
Effect of TcdA of *Clostridium difficile* and alanyl-glutamine on TUNEL cells/mm^2^ percent. The IEC-6 cells were treated for 24 h medium alone (control), TcdA (10 ng/mL), TcdA + Ala-Gln (treated with TcdA 10 ng/mL and Ala-Gln 10 mM), and Ala-Gln 10 mM. *P < 0.05 compared to control. ^#^ *P* < 0.05 compared to TcdA 100 ng/mL.

 As shown in Figure 5, exposure to TcdA at 10 ng/mL for 24 h increased the percentage of TUNEL-positive cell to 15% (*P* < 0.05, compared to control). Supplementation with Ala-Gln at 10 mM for 24 h simultaneously with TcdA exposure decreased the percentage of TUNEL-positive cell by 61.4% compared to TcdA alone (*P* < 0.05). Effect of TcdA and Ala-Gln on cell proliferation.

### 3.4. Indirect Evaluation of Cell Proliferation by WST Assay

**Figure 6:**
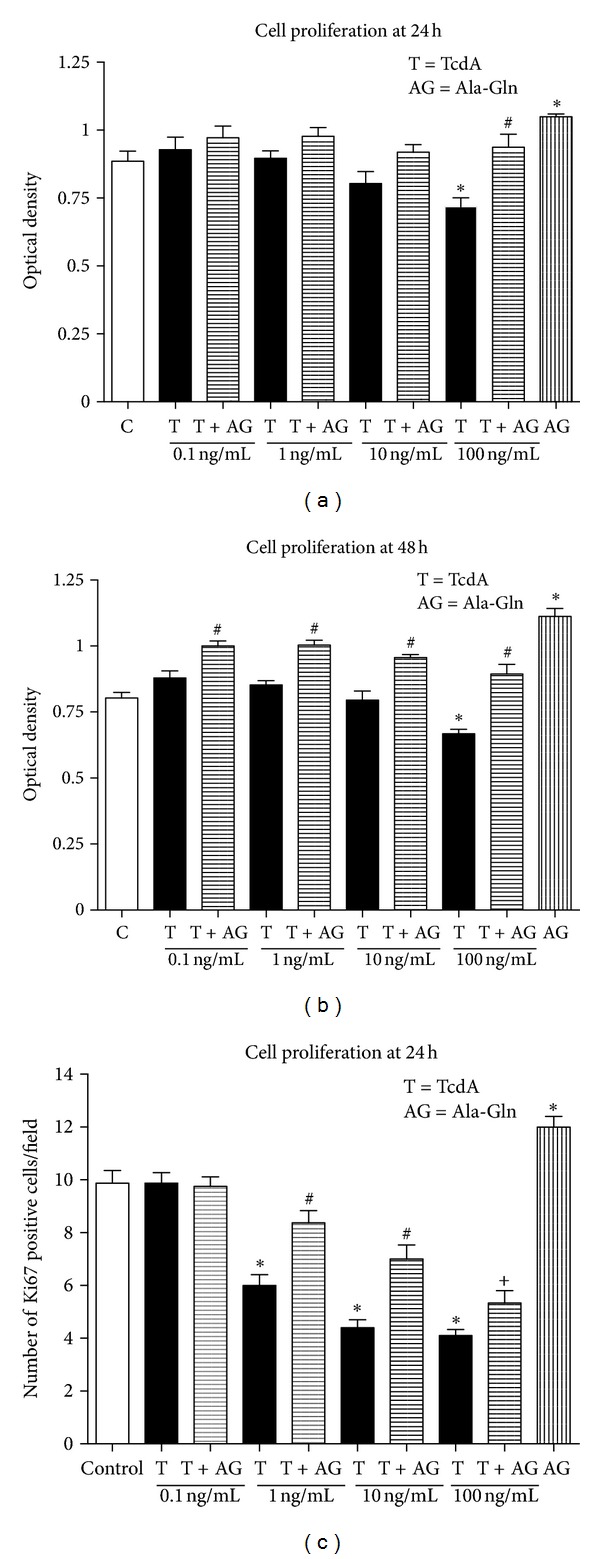
Effect of TcdA of *Clostridium difficile* and glutamine and alanyl-glutamine on cell proliferation. (a) The IEC-6 cells were exposed to TcdA (at 0.1, 1, 10, or 100 ng/mL) and supplemented or not with Ala-Gln at 10 mM for 24 h. (b) The cells were exposed to TcdA (at 0.1, 1, 10, or 100 ng/mL) and supplemented or not with Ala-Gln at 10 mM for 48 h. The WST absorbance was measured using an ELISA microplate reader at 450 nm (reference range 420–480 nm). (c) The cells treated with TcdA for 24 h, the number of cells positive for Ki67. *P < 0.05 compared to control. ^#^ *P* < 0.05 compared to TcdA by one-way ANOVA. ^+^ *P* < 0.05 compared to TcdA by unpaired Student's *t*-test.

As shown in Figure 6(a), exposure to TcdA at 100 ng/mL for 24 h decreased cell proliferation by 17.0% (*P* < 0.05, compared to control). Supplementation with 10 mM of Ala-Gln prevented the antiproliferative effect of TcdA (*P* < 0.05). Similarly, as seen in Figure 6(b), after 48 h of exposure with TcdA at 100 ng/mL decreased proliferation by 20.7%, respectively (*P* < 0.05). Supplementation with Ala-Gln significantly prevented the antiproliferative effect of TcdA at 100 ng/mL (*P* < 0.05 by one-way ANOVA).

### 3.5. Direct Evaluation of Cell Proliferation by Ki67 Assay

 As shown in Figure 6(c), exposure to TcdA for 24 h at 1 ng/mL, 10 ng/mL and 100 ng/mL decreased cell proliferation by 39.3%, 55.4%, and 58.4%, respectively (*P* < 0.05, compared to control group). Supplementation with Ala-Gln at 10 mM significantly prevented the antiproliferative effect of toxin A at 1, 10 (*P* < 0.05 by one-way ANOVA) and at 100 ng/mL (*P* = 0.02 by unpaired Student's *t*-test).

### 3.6. Effect of TcdA and Ala-Gln or Gln Supplementation on Distribution and Expression of f-Actin and RhoA

**Figure 7:**
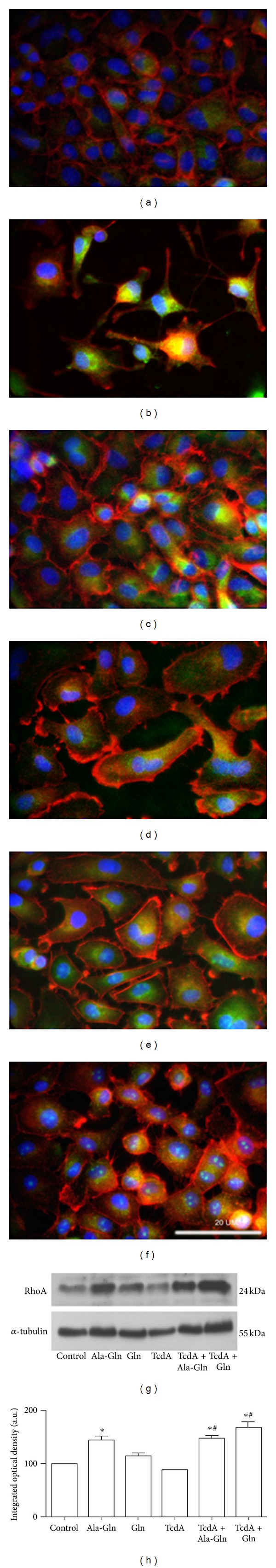
Fluorescent microscopy on distribution of f-actin fibers and RhoA and immunoblotting analysis of RhoA for the effect of TcdA of *Clostridium difficile *and the effect of micronutrients. The cells were treated with TcdA at 10 ng/mL for 24 h and supplemented or not with 10 mM of Ala-Gln or Gln and incubated with rhodamine-phalloidin, FITC-RhoA, and DAPI. The IEC-6 cells were divided into 6 groups: control (a); treated with TcdA at 10 ng/mL for 24 h (b); treated with 10 mM of alanyl-glutamine without TcdA (c); treated with 10 mM of glutamine alone without TcdA (d); treated with TcdA at 10 ng/mL for 24 h and supplemented with 10 mM alanyl-glutamine (e); treated with TcdA at 10 ng/mL for 24 h and supplemented 10 mM of glutamine (f). Immunoblotting was performed to evaluate the expression of RhoA (g and h). The quantification was done comparatively defaulting protein *α*-tubulin. *P < 0.05 compared to control. ^#^ *P* < 0.05 compared to TcdA.

As demonstrated in Figure 7(b), incubation with TcdA for 24 h caused change in F-actin distribution (red staining) causing cytoskeleton collapse around the nucleus of the IEC6 cells in the monolayer. In addition, TcdA-treated cells show decreased staining of actin filaments beyond the plasma membrane and loss of adherence. At the same time, TcdA-treated cells show RhoA (green staining) concentrated close to the nucleus overlapping the f-actin fibers as can be seen by orange staining. Additionally, as seen with DAPI staining, TcdA caused nuclear fading and nuclear pyknosis. The control cell monolayer (incubated in media without Gln) showed more homogeneous actin distribution with actin bundles attached to the plasma membrane and RhoA homogeneously distributed around the cytoplasm (Figure 7(a)). Supplementation with both Gln and Ala-Gln partially reverted the changes described in cells treated with TcdA alone (Figures 7(e) and 7(f)), showing preservation of F-actin cytoskeleton and a more organized intracellular actin network preserving actin bundles attached to the plasma membrane. The preservation of cytoskeleton was associated with increased expression of RhoA in the cytoplasm (green staining) which was confirmed by increased RhoA protein expression as detected by Western Blotting (Figures 7(g) and 7(f)). In cells not treated with TcdA, supplementation with 10 mM of Ala-Gln and Gln showed robust and organized intracellular actin network associated with increased expression of RhoA protein (Figures 7(c) and 7(d)).

## 4. Discussion

In this study, we used different microscopic approaches in order to demonstrate, in detail, the effect of TcdA in intestinal epithelial cell morphology and their association with cell death and proliferation and to evaluate the protective effect of Gln and Ala-Gln on TcdA-induced cell damage. High-resolution advanced microscopic techniques such as confocal laser fluorescence microscopy, atomic force microscopy, and scanning electron microscopy are capable of providing detailed morphological features and cytoskeletal information. In particular, AFM, besides its high-resolution capabilities, provides advantage over traditional microscopic techniques because it is not restricted to cell morphology, cytoskeletal elements, or organelles, but also provides quantitative information on cell volume, area, and height. We showed that TcdA, in higher concentration, caused dramatic changes in cell morphlogy as assessed by AFM associated with significant decrease in both cell volume and area and increase in cell height, as a result of shrinkage of the cell associated with aggregation of cellular material around the nucleus. In addition, TcdA caused disruption of the nuclear envelope and chromatin condensation, as early as, with 1 h of incubation. Most of these morphological changes were confirmed by SEM. 

Disruption of cytoskeleton was seen by confocal microscopy. The organization of the cytoskeleton with its rapid assembly and disassembly has important role in motility, guidance, and adhesion of cells [35]. In intestinal epithelial cells, involved in maintenance of the barrier function, cytoskeletal disruption by TcdA may damage tight junctions and cause failure of focal contact formation, possibly leading to exposure of luminal pathogens to immune cells in the lamina propria [36, 37]. TcdA induces monoglycosylation of Rho, Rac, and Cdc42 at threonine 37, preventing Rho family proteins from participating in the formation of actin filaments [38]. This mechanism is believed to be the main cause for the cell morphological changes induced by TcdA, which can be a consequence of the cytoskeleton collapse as seen by confocal microscopy. Rho glycosylation by TcdA results in the disappearance of actin cables, peripheral membrane ruffling, filopodial extensions, and the disorganization of focal complexes, and ultimately resulting in complete loss of cell shape, that is, cell rounding as seen here [39, 40].

Furthermore, TcdA increased cell death as measured by TUNEL assay. The increase in TUNEL-positive cells indicate cell death, and this data, associated with the morphological feature showed here, suggest cell death by apoptosis, as reported previously [23, 25, 26], which could contribute to the disruption of intestinal epithelial barrier leading to the severe inflammatory response seen in response to *C. difficile* infection. It has been suggested that necrosis may be more important than apoptosis in *C. difficile* pathogenesis, since necrosis involves the release of cytoplasmic contents and induction of an immune response, leading to local inflammation [16]. However, it is well known that, depending on the intensity and duration of the stimulus, the damage can start with apoptosis and progress to necrosis. 

Nam et al. gave new insight into the mechanisms involved in the rapid cell rounding induced by TcdA [19]. Their group demonstrated that in addition to the effect in inactivation of Rho family proteins and actin disaggregation, TcdA induces tubulin deacetylation and microtubule depolymerization through HDAC6 cytosolic tubulin deacetylase. Microtubule instability is critical to cell shape [41], cell movement [42], intracellular transport of organelles [43], and the separation of chromosomes during mitosis [40], which could explain the decreased proliferation induced by TcdA showed here.

Although the TcdA has well-known effect on Rho A glycosylation and inactivation, we did not observe any significant differences in RhoA concentration following TcdA exposure as compared to control. However, supplementation with Gln and Ala-Gln increased RhoA concentration even in the presence of TcdA, which could, at least in part, explain the protective effect of these micronutrients on TcdA-induced morphological changes, cell death, and inhibition of proliferation. These finding are in accordance with previous studies which showed that Gln and Ala-Gln prevented the inhibition of cell migration, apoptosis, and the initial drop in transepithelial resistance induced by TcdA [23]. These effects may be explained by the partial preservation of the cytoskeleton as visualized here by immunofluorescence, probably as a consequence of the increase of RhoA expression. Furthermore, Gln and Ala-Gln have been reported previously to inhibit the apoptosis of T84 cells by preventing caspase 8 activation and to reduce TcdA-induced intestinal secretion and epithelial disruption [26]. Since cytoskeleton is involved in separation of chromosomes during mitosis, it is not surprising that its preservation by the micronutrients leads to increased proliferation even in the presence of TcdA. Bilban et al. showed that the main signaling pathway for cell survival is the activation of HSPs, whose action is regulated by Gln [44]. Gabai and Sherman showed, in some models of disease, that the protective effect of Gln requires induction of hemoxigenase-1 (HO-1) [45]. Our research group suggested that the pathway HO-1/carbon monoxide has a significant protective effect when there is injury caused by the TcdA,including decreased neutrophilic infiltrate in the mucosa. Studies by several authors showed that Gln and its stable derivative Ala-Gln have effective actions on cell proliferation, apoptosis, and protein synthesis [46, 47]. Additionally, we observed that supplementation with Ala-Gln or Gln alone increased RhoA protein concentration. The overproduction of RhoA may partially explain the protective effect of glutamine and alanyl-glutamine in the cytotoxicity induced by toxin A.

## 5. Conclusions

To the best of our knowledge, this is the first study conducted evaluating the effect of *C. difficile* toxins and Ala-Gln and Gln treatment on cell morphology using AFM. These AFM images substantiate the confocal study of the effect of TcdA on intestinal epithelial cell lines. Moreover, the aberration in intestinal epithelial cell morphology and actin filament organization mediated by TcdA may, at least, partially account for the severe intestinal mucosal disruption found in *C. difficile*-induced disease.
